# Agarose stamped method: a simple and customizable immobilization technique for zebrafish larvae

**DOI:** 10.3389/fnbeh.2025.1692708

**Published:** 2025-10-07

**Authors:** John Jutoy, Hossein Mehrabi, Pushkar Bansal, Erica E. Jung

**Affiliations:** ^1^Department of Mechanical Engineering, University of Illinois Chicago, Chicago, IL, United States; ^2^Department of Pharmacology and Toxicology, The University of Utah, Salt Lake City, UT, United States

**Keywords:** zebrafish larvae, agarose stamping, immobilization, behavioral assays, neuroimaging, high-throughput imaging

## Abstract

Standardized immobilization of zebrafish larvae is crucial for consistent behavioral assays such as optokinetic response, feeding, and tail-movement analyses, but traditional agarose embedding methods remain labor-intensive and variable. We developed the Agarose Stamped Device (ASD), a low-cost platform that imprints larva-sized wells into agarose, enabling rapid and reproducible alignment of multiple larvae while preserving viability. Customizable designs permit immobilization while maintaining eye, mouth, or tail freedom—achieved far more easily than with traditional embedding and post-processing. We demonstrate that the ASD sufficiently stabilizes larvae for high-resolution eye tracking, feeding assays, and tail-movement analyses. By combining standardized positioning with behavioral flexibility, the ASD broadens the range of feasible zebrafish experiments and lowers barriers to high-throughput behavioral neuroscience.

## 1 Introduction

Larval zebrafish (*Danio rerio*) have become an indispensable model for neuroscience and behavioral research due to their genetic tractability, small size, and optical transparency (Pulak, 2016; Fetcho and Liu, 1998; Schweitzer et al., 2012). Foundational studies established that zebrafish larvae can model complex biological processes from neurodevelopment to behavior, enabling high-throughput drug screens and neurobiological assays in a whole-organism context (Fetcho and Liu, 1998; Zon and Peterson, 2005). Early behavioral assays often employed free-swimming larvae in multi-well plates or Petri dishes to observe locomotor responses and drug effects. However, while these open-well methods are straightforward, they offer limited experimental control—stimulus delivery and behavioral readouts tend to be qualitative and inconsistent (Peimani et al., 2018). As the field has advanced, there is a growing need for methods to restrain or position larvae in reproducible orientations for high-resolution imaging and precise stimulus presentation, without compromising the animal's viability or normal behavior.

The conventional approach for immobilizing zebrafish larvae is to embed them in low-melting-point agarose. This simple, low-barrier method provides adequate immobilization for microscopy or targeted stimulation and has been widely used in neurobehavioral experiments. Unfortunately, agarose embedding presents several well-recognized challenges. Fully encasing a larva in agarose can impede its growth or physiological processes and has been reported to induce stress (e.g. cardiac strain, tissue necrosis) when maintained for prolonged periods (Huemer et al., 2017a,b). Standard agarose mounts also solidify rapidly, giving researchers only a brief window to orient each larva under the microscope. Manipulating larvae into position before the gel sets require considerable skill and risks physical damage to the specimen (Lisse et al., 2015; Kamei et al., 2010). Moreover, once solidified, the agarose may need to be partially removed to allow certain behaviors or growth to proceed, a process that itself can injure the larva (Lisse et al., 2015). These drawbacks limit the utility of plain agarose for long-term behavioral studies or experiments requiring frequent interventions.

To overcome such issues, researchers have explored both low-barrier and high-barrier entry methods for zebrafish larval immobilization. On the low-barrier end, simple innovations like casting agarose wells or “divots” in advance have been used to guide larval positioning (Huemer et al., 2017a). For example, ridged agarose molds can hold larvae in arrayed rows, reducing the hands-on alignment needed before imaging (Huemer et al., 2017a). Such approaches retain the low cost and accessibility of agarose while improving consistency. However, they still involve manual preparation and do not completely resolve limitations in long-term viability or access to the specimen. On the high-barrier end, advanced microfluidic and engineered devices have recently been developed to precisely position and hold zebrafish larvae. Notably, Beebe and colleagues introduced the ZEBRA (Zebrafish Entrapment by Restriction Array) microfluidic device as an agarose-free mounting technique that uses only a pipette for loading larvae into preset channels (Bischel et al., 2013). This and other “fish-on-a-chip” systems provide fine spatial control, allowing parallel orientation of multiple larvae and easy delivery of stimuli or reagents via built-in ports (Bischel et al., 2013). Such high-end devices have enabled new experimental paradigms—for instance, microfluidic platforms can expose larvae to controlled flow or electric fields to study rheotaxis and electrotaxis behaviors with quantitative precision (Peimani et al., 2018). They have also been applied to high-throughput drug screens and whole-brain activity mapping *in vivo* (Bansal et al., 2021). The downside is that these setups often require specialized fabrication and can pose a higher barrier to entry for many labs. Indeed, one persistent challenge with microfluidic larval holders is limited throughput; handling and imaging larvae one-by-one (or only a few at a time) can be time-consuming compared to traditional multi-well assays (Khalili et al., 2022). Recent advancements are beginning to address this by scaling up device capacity (e.g. from single-larva to four-larva chips) and streamlining loading procedures (Khalili et al., 2022), but the complexity and cost of microdevices still motivate the search for simpler alternatives.

In this context, we introduce the Agarose Stamped Device (ASD) an expansion on the work on agarose molds and stamps for zebrafish larval alignment and immobilization for behavioral experiments. The ASD bridges the gap between low-barrier agarose embedding and higher-barrier microfluidic devices. It consists of a singular or patterned stamp that imprints one or multiple shallow larva-sized wells into agarose, creating a ready-to-use template for positioning and immobilizing larvae. Using the ASD, a researcher can immobilize dozens of larvae in one step by simply placing them into the stamped wells, where they can be carefully guided into the wells. This approach offers several key advantages over existing methods. First, it drastically reduces handling time and user skill required—there is less effort required to individually align and position each larva. Second, because each well is identical, the larvae are uniformly aligned (for example, all facing the same direction and lying on the same focal plane), which improves imaging consistency and assay reproducibility. Third, the stamped agarose substrate leaves the larvae partly exposed to the surrounding solution, creating an open system that permits easy access for experimental manipulations. For instance, drugs or stimuli can be added to the medium at any time, and specific body parts (e.g. the tail or eyes) can be targeted for stimulation or recording, something not possible when a larva is fully embedded in agarose. Importantly, the gentle physical restraint provided by the ASD is sufficient to hold larvae in place for microscopy or behavioral monitoring, while minimizing stress and allowing normal physiological functions (heartbeat, blood flow, neural activity) to continue unimpeded.

ASD builds upon the recent works that larvae can be stably maintained and imaged within a stamped agarose well for prolonged periods of time (Huemer et al., 2017a; Alessandri et al., 2017; Copper et al., 2018; Kleinhans and Lecaudey, 2019), underscoring the method's applicability across various developmental stages. However, these prior systems such as the UniverSlide (Alessandri et al., 2017) were primarily developed for broader microscopy applications rather than for zebrafish larva-specific behavioral research. Commercially, stamping agarose as a method was deemed so useful at positioning larvae for microscopy that it was turned into a product (Stampwell Z, Idylle, France), selling for hundreds of dollars. Others sought to make these molds more accessible by making them 3D-Printable (Kleinhans and Lecaudey, 2019). Similarly, we aimed to make the ASD stamps as simply and as accessible as possible such that a hobby desktop resin printer like the Elegoo Mars 2 (Elegoo, China) would be able to make them.

Where our work differs from these earlier devices is our systematic analysis of device parameters critical for larval behavior, such as optimized agarose concentration, cavity geometry for stable alignment, or specialized configurations (e.g., free-tail, free-eye, or free-mouth) that preserve natural motor outputs. Moreover, these works did not provide quantitative validation of larva alignment or address cost and accessibility with documentation, step by step instructions, and editable CAD files. In contrast, the ASD presented here was explicitly designed and validated for zebrafish larval neuroscience and behavioral assays. By combining tunable geometries, quantitative alignment metrics, and a fabrication workflow that emphasizes cost efficiency and adaptability, the ASD advances agarose-based immobilization into a practical, reproducible, and low-cost alternative tailored for experimental paradigms that require both stability and behavioral fidelity.

Furthermore, ASD also opens new opportunities for zebrafish behavioral experiments. It enables high-content analyses where many larvae are immobilized in parallel—for example, drug screening assays can treat multiple aligned larvae with compounds and directly compare their responses under the same optical field. Likewise, neurobehavioral studies requiring simultaneous imaging of neural activity in several animals (such as brain calcium imaging during sensory stimulation) can benefit from the standardized orientation and stability the ASD provides.

In this work, we validate the ASD's effectiveness in reliably immobilizing larvae in place and maintaining consistent alignment across individuals. We demonstrate that larvae mounted with the ASD remain properly aligned and exhibit expected behaviors or responses, indicating that the device imposes minimal adverse effects. Overall, the Agarose Stamped Device offers a convenient and reproducible immobilization strategy that addresses many challenges of conventional agarose embedding. We propose that the ASD will facilitate a wide range of zebrafish larval assays—from developmental imaging to behavioral neuroscience—by providing an accessible yet robust means of larval immobilization and alignment. The following sections detail the design of the ASD, its immobilization performance, and example applications that highlight its utility in zebrafish behavioral research.

## 2 Materials and methods

### 2.1 Developed protocols

The following protocols outline the standardized fabrication and application of agarose stamping devices for zebrafish larval immobilization. This method enables the rapid and reproducible creation of larva-shaped cavities in agarose using customizable 3D-printed stamps. The protocol is divided into three key stages: CAD-based stamp design, agarose device fabrication, and larval positioning for experimental use. Each step is structured to ensure dimensional fidelity, minimize handling stress, and support a range of experimental paradigms including imaging, behavioral assays, and screening. By formalizing these procedures, the agarose stamping methodology provides a consistent, accessible, and high-throughput alternative to traditional embedding techniques.

#### 2.1.1 CAD design and stamp preparation

**Figure 1:**
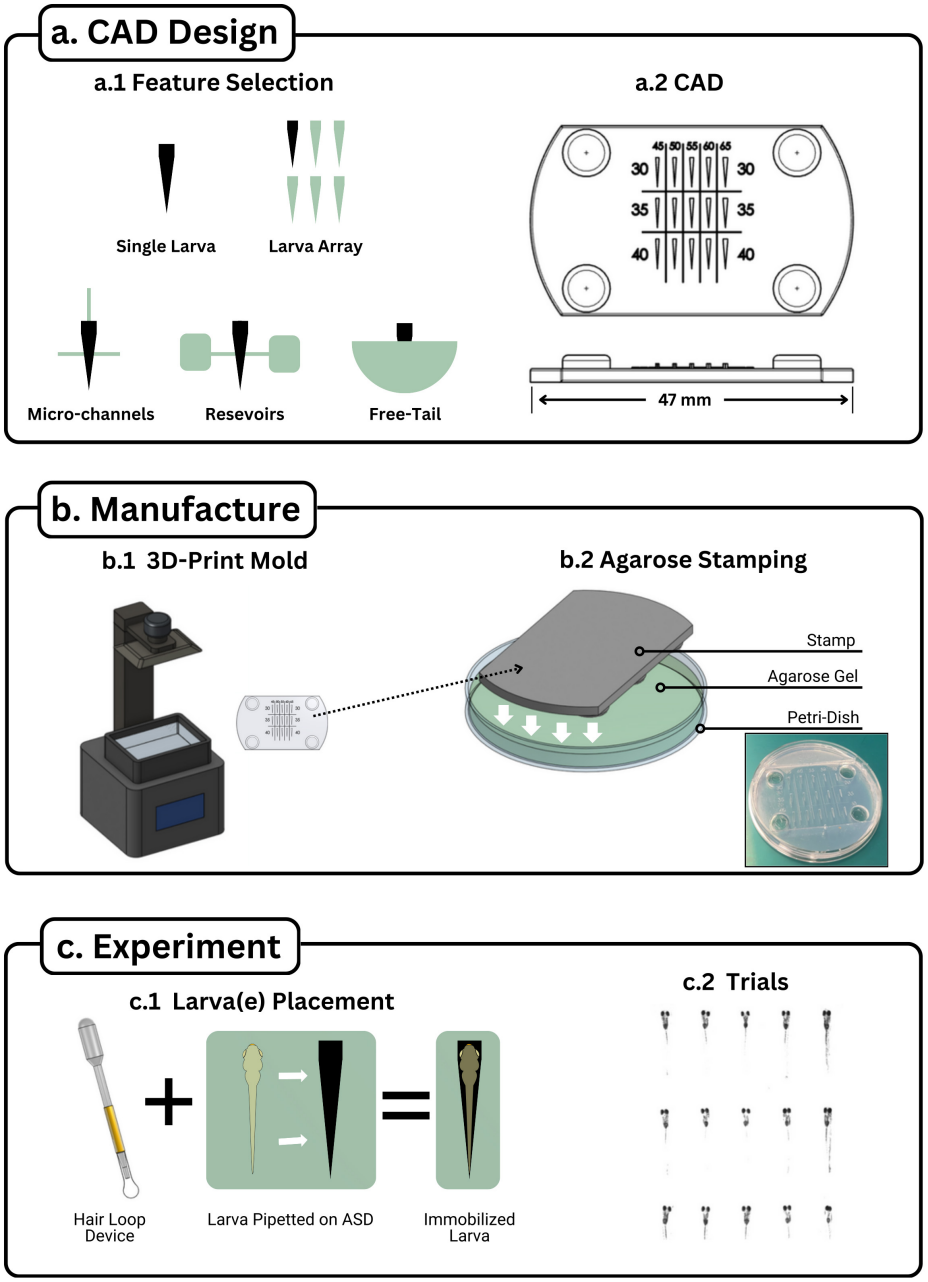
Agarose stamping workflow. The Agarose Stamping Workflow visualized into three sections: **(a)** CAD Design, **(b)** Device Fabrication, and **(c)** Device Utilization. (a.1) Various features can augment the single stamp setup to accommodate requirements for experiments requiring high throughput, fluid channels, fluid reservoirs, and free tail configurations. (a.2) Once features have been chosen, they can be integrated together through CAD software to create a stamp model. A high throughput device is displayed with values displaying changes in two parameters: increasing head width among the columns and increasing head length among the rows. Note that four extruded circles were included to act as a spacer from the agarose. (b.1) The model can be printed with a resin printer that has at least 50 μm resolution. (b.2) The printed stamp is placed on a petri dish and is submerged up to the stamp's base with heated liquid agarose. (c.1) Larva is placed near the cavity (negative larva shape that was made with the stamp) via disposable pipette. The larva is maneuvered into the hole by moving it with a hair loop device, ensuring that the head of the larvae matches the cavity. (c.2) High throughput device with 15 larvae immobilized and ran through a visual stimulation trial.

1. Define Experimental Requirements
Determine the desired features of the agarose stamp based on the specific experimental application. Possible features include (Figure 1a.1):

° Single-larva cavities for individualized assays.
° Multi-larva arrays for high-throughput screening.
° Microchannels for fluidic connectivity.
° Reservoirs for sustained hydration.
° Free-tail or free-eye configurations for behavioral assays involving movement.

2. Design the Stamp in CAD Software
Construct a three-dimensional model incorporating the selected features using CAD software. Ensure that the geometry accommodates the anatomical dimensions of the larva and supports consistent positioning (Figure 1a.2). Modifiable CAD files of stamps have been provided in repositories inside Supplementary File 1.
3. Export for 3D Printing
Export the finalized CAD model as a stereolithography (.STL) file, suitable for 3D printing.
4. 3D Print the Stamp
Fabricate the stamp using a resin-based 3D printer with a minimum resolution of at least 50 μm (Figure 1b.1). Proper post-processing including isopropyl alcohol bath and ultraviolet curing is required to ensure dimensional accuracy and structural stability.

#### 2.1.2 Agarose device fabrication

5. Prepare the Petri Dish Assembly
Place the 3D-printed stamp inside a standard Petri dish. Orient the stamp such that the imprinting surface faces into the Petri dish.
6. Pour Molten Agarose
Pour molten 1.5% agarose solution into the Petri dish, ensuring that the solution surrounds the stamp and reaches the upper surface of the stamp.
7. Solidify the Agarose
Allow the agarose to cool and solidify completely (~15 min) without disturbance.
8. Remove the Stamp
Carefully extract the stamp by lifting vertically to avoid disrupting the agarose cavities. If needed, a fine blade and tweezers may be used to gently free the stamp from surrounding agarose (Figure 1b.2).

#### 2.1.3 Larval placement and experimental setup

9. Introduce Larvae to the Stamped Device
Using a disposable pipette, transfer zebrafish larvae into the agarose-stamped Petri dish, positioning each near its designated cavity.
10. Position the Larvae
Employ a hair loop tool to gently guide each larva into its respective cavity. Confirm that the head and body are correctly aligned within the cavity without causing physical strain (Figure 1c.1).
11. Commence Experimental Protocols
Once all larvae are immobilized and oriented, proceed with the intended experimental procedure (Figure 1c.2).

### 2.2 Zebrafish lines, maintenance, and breeding

Wild-type AB strain zebrafish (ABWT) larvae at 7 days post-fertilization (dpf) were used in all experiments outside of screening. Embryos were bred and raised in the zebrafish facility at the University of Illinois at Chicago (UIC). Adult fish were housed in a recirculating aquatic system (Aquaneering, Inc., San Diego, CA) under standardized conditions, including a 14-h light/10-h dark photoperiod and a controlled temperature of 27 °C. Larvae were maintained in the facility until they reached 7 dpf, at which point they were transferred to the experimental lab for analysis. Prior to experiments, all larvae were kept in standard system water and not fed, ensuring consistency across experimental conditions.

Several transgenic lines were used for specific experiments. For screening experiments, SAIGFF213A; UAS:GCaMP6s double transgenic zebrafish embryos utilized for fluorescence screening. The SAIGFF213A line expresses Gal4FF, a modified form of the Gal4 transcription factor, in a defined subset of spinal neurons, including the caudal primary (CaP) motor neurons (Muto et al., 2011). When crossed with the UAS:GCaMP6s line, these embryos express the calcium indicator GCaMP6s specifically in these neurons. At 6 days post-fertilization (dpf), positive larvae were identified by the presence of distinct fluorescence in the spinal cord and motor neurons upon blue light (470 nm) illumination, whereas negative siblings lacked such fluorescence. This genetic approach enabled selective visualization of spinal neuronal populations. Feeding experiments utilized both ABWT larva and the transgenic line Tg(AgRP1:ChR2-Kaede) in which AgRP1 neurons can be optogenetically stimulated.

All procedures involving zebrafish were conducted in accordance with institutional and national ethical guidelines. Protocols were reviewed and approved by the Institutional Animal Care and Use Committee (IACUC) at UIC under protocol number 24-115. The institution is accredited and holds Animal Welfare Assurance Number D16-00290 (A3460.01), registered with the Office of Laboratory Animal Welfare (OLAW), NIH.

### 2.3 Stamp and device fabrication

The stamps for the Agarose Stamped Devices (ASDs) were designed in CAD software (Onshape, Onshape Inc., USA) to approximate the body profile of 7 dpf zebrafish larvae. Design variations incorporated free-tail, free-eye, and arrayed geometries, depending on experimental requirements. Models were exported as stereolithography (STL) files and fabricated using a resin-based stereolithography printer (Elegoo Mars 2 Pro, Elegoo Inc., China) at a resolution of 50 μm. Following printing, each stamp was rinsed in 75% isopropyl alcohol (IPA) to remove uncured resin and subsequently cured under 405 nm ultraviolet light for 1 min per side using a portable UV curing chamber (Elegoo Mercury Plus V2.0, Elegoo Inc., China).

To generate agarose stamps, agarose (A6877, Sigma-Aldrich, USA) solutions of 1.5%, 2%, and 3% (w/v) were prepared in a glass bottle and heated in a microwave until fully liquefied. The molten agarose was poured into a 47 mm petri dish (PD-1, Advantec MFS, USA) containing the 3D-printed stamp with the stamping features oriented into the petri dish. The agarose was allowed to solidify at room temperature for 15 min before the stamps were carefully removed using fine-tipped tweezers. To avoid tearing, excess agarose in contact with the base of the stamp was separated with a blade prior to lifting.

### 2.4 Video processing

Video recordings for larval eye and tail tracking were acquired using a microscope (SM-1 Series, Amscope, USA) paired with a microscope camera (XiQ MQ013MG-ON-S7, XIMEA GmbH, Germany). All recordings were captured at frame rates between 30 and 60 frames per second to ensure adequate temporal resolution for kinematic analysis.

For eye tracking, a custom Python-based computer vision pipeline was employed - derived from our work in Zebrafish Larva Interface (Jutoy et al., 2025). The software extracted eye contours via thresholding and ellipse-fitting procedures applied to all larvae in an image, from which eye data: angular positions, center of mass location, area was computed.

Tail kinematics were extracted using ZebraZoom (Mirat et al., 2013), an established open-source tool optimized for zebrafish motion tracking. Key points along the larval midline were automatically annotated, and tail deflection angles were computed for a tail-free, eye free larva with rotating visual stimuli displayed ventrally through an LCD screen (5inch LCD Display-B, ELECROW, China).

Suction tracking was conducted following the procedure described by Mehrabi et al. (2025), in which optogenetic activation of hypothalamic AgRP neurons in transgenic zebrafish larvae was shown to increase food intake.

### 2.5 Alignment metrics

Zebrafish larvae exhibit size variations based on factors such as age, water temperature, stocking density, water quality, genetic background, and food availability (van de Pol et al., 2021; Singleman and Holtzman, 2014). In this study, we standardized these parameters and used 7 days post-fertilization (dpf) larvae. To perform various experiments, it is necessary to immobilize the larvae within a slot while maintaining their viability and natural behavior. The slot dimensions had to be optimized to ensure that the larvae are neither excessively constrained, which could cause damage or alter their physiological state, nor too loosely positioned, which would allow uncontrolled movement.

To determine the optimal slot dimensions, we varied both head width and slot depth. A smaller head width and increased depth resulted in a tighter fit, imposing greater restriction, while a larger head width and shallower depth provided greater freedom of movement. We examined head widths ranging from 0.325 mm to 0.725 mm in 0.1 mm increments and slot depths from 0.3 mm to 0.7 mm with the same increment. When evaluating the effect of head width, the depth was fixed at 0.5 mm, and when analyzing depth, the head width was fixed at 0.525 mm. For each head width and depth combination, nine samples were analyzed, resulting in a total of 90 samples across all conditions.

After positioning the larvae in the slot, their alignment and stability were recorded from a top-view perspective at 35 frames per second (fps) for 10 minutes. The device design incorporated water reservoirs to maintain a stable water level within the slots and microchannels, preventing dehydration or stress over time.

#### 2.5.1 Alignment and stability quantification

The alignment and stability of each larva were assessed based on eye positioning, as the eyes serve as clear and quantifiable markers. A well-aligned larva exhibits symmetrical eyes from the top view, and a stable larva maintains this symmetry over time. Misalignment or instability can manifest as:

1. Rotation along the anteroposterior (AP) axis, causing asymmetry in eye positioning.
2. Mediolateral (ML) displacement, resulting in lateral eye shifts along the *x*-axis.
3. Anteroposterior (AP) displacement, leading to movement along the *y*-axis.

To quantify these misalignments, we analyzed eye area differences and *x*/*y* coordinate variations over time.

#### 2.5.2 Misalignment measurement: eye area differences

When the larva rotates along the AP axis, the eyes become asymmetric, and the absolute difference in eye areas becomes nonzero. A higher value indicates greater misalignment.

For each frame *i* in a sample, we calculated:


$$\begin{array}{l} \Delta A_{i}=|A_{right,i}-A_{left,i}|. \end{array}$$

Where *A*_*right, i*_ and *A*_*left, i*_ are right and left eye area in frame *i*.

The mean eye area per sample was determined as:


$$\begin{array}{l} A_{mean}=\frac{\sum_{i=1}^{n}\left(A_{right,i}+A_{left,i}\right)}{2n}. \end{array}$$

The normalized eye area difference was then computed as:


$$\begin{array}{l} \Delta A_{norm}=\;\frac{\Delta A}{A_{mean}} \end{array}$$

To assess variation across frames, we computed the standard deviation of the eye area differences:


$$\begin{array}{l} \sigma_{\Delta A}=Standard\;Deviation\left(\Delta A\right). \end{array}$$

For each experimental condition, we analyzed 45 samples and determined:


$$\begin{array}{l} A_{\max}=\max\left(A_{mean}\right).\\ \sigma_{\max}=\max\left({\sigma \;}_{\Delta A}\right). \end{array}$$

#### 2.5.3 Handling overlapping eyes

If the eyes overlap due to misalignment, the algorithm cannot distinguish between them, and the eye area measurement is assigned NaN. To quantify this, we calculated the NaN ratio:


$$\begin{array}{l} R_{NaN}=\;\frac{Number\;of\;NaNs\;in\;\Delta A}{Total\;number\;of\;frames}. \end{array}$$

#### 2.5.4 Distinguishing misalignment from instability

Eye area differences may result from misalignment, which is accounted for in the normalized mean, or instability, which is accounted for in the normalized standard deviation. To separate these effects, we computed:


$$\begin{array}{l} \Delta A_{norm\;mean}=\;\frac{mean\;\left(\Delta A_{norm}\right)}{A_{\max}}.\\ \sigma_{norm}=\;\frac{SD\;\left(\Delta A_{norm}\right)}{\sigma_{\max}}. \end{array}$$

Finally, the alignment score was computed as:


$$\begin{array}{l} S_{align}=\;\left(1\;-\;\frac{w_{1}\;.\;\Delta A_{norm\;mean}+w_{2}\;.\;\sigma_{norm}}{2}\right)\;.\;\left(1\;-R_{NaN}\right). \end{array}$$

Where *w*_1_ and *w*_2_ are weights that sum to 1. We used *w*_1_ = *w*_2_ = 0.5, and the results remained consistent across different values.

#### 2.5.5 Instability measurement: eye coordinate variations

When the larva experiences ML or AP displacement, the *x*/*y* coordinates of the eyes fluctuate over time. The same analysis used for eye area differences was applied, with the exception that the average was not considered, only the standard deviation:


$$\begin{array}{l} w_{1}=0,\;w_{2}=1. \end{array}$$

Instead of using eye area differences, we computed eye coordinate averages:


$$\begin{array}{l} X_{avg,\;i}=\frac{x_{right,i}+x_{left,i}}{2}\;for\;all\;i. \end{array}$$

The sample mean for x-coordinates was:


$$\begin{array}{l} X_{mean}=\frac{\sum_{i=1}^{n}X_{avg,\;i}}{n}. \end{array}$$

Similarly, for y-coordinates:


$$\begin{array}{l} Y_{avg,\;i}=\frac{y_{right,i}+y_{left,i}}{2}\;for\;all\;i.\\ Y_{mean}=\frac{\sum_{i=1}^{n}Y_{avg,\;i}}{n}. \end{array}$$

And the rest of the process follows the same methodology as the eye area differences.

## 3 Results

### 3.1 Protocol development

A standardized workflow for the Agarose Stamped Device (ASD) was established, consisting of CAD-based stamp design, agarose device fabrication, and larval placement. CAD designs incorporated features such as single-larva cavities, multi-larva arrays, reservoirs, and microchannels, with customizable geometries tailored to specific experimental needs. Resin-based 3D printing (≥50 μm resolution) reliably produced stamps that, when pressed into molten agarose, yielded cavities consistent with larval morphology. The use of a hair-loop tool for placement minimized handling stress and ensured correct orientation within cavities.

### 3.2 Optimal agarose concentration

**Figure 2:**
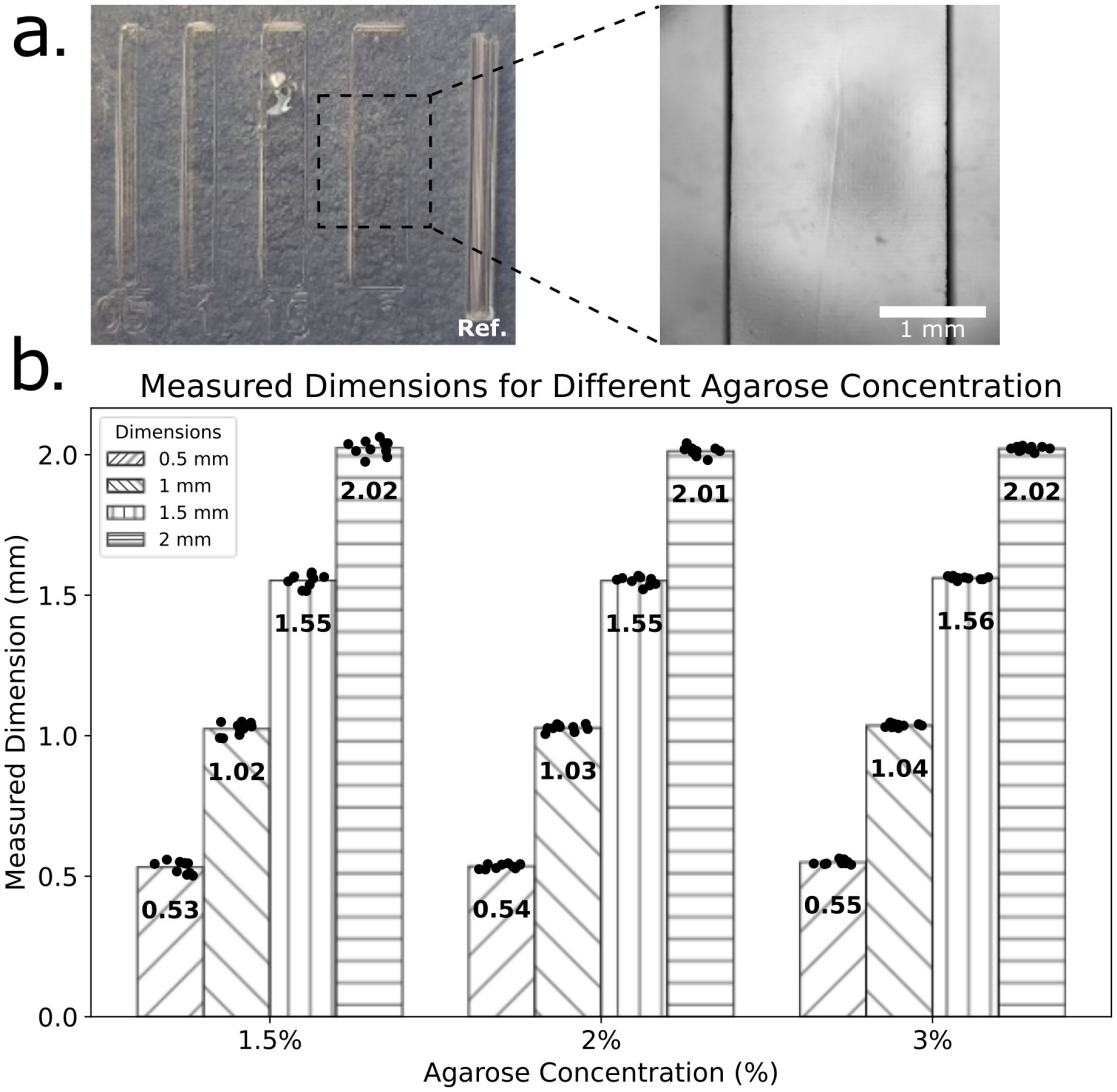
Evaluation of feature dimension fidelity in Agarose Stamped Devices (ASD). **(a)** Microfabricated features with nominal widths of 0.5, 1, 1.5, and 2 mm imprinted in agarose using a 3D-printed stamp. A 1 mm reference feature is included in each set for dimensional calibration. A magnified view of the 2 mm feature is shown, with a scale bar of 1 mm for reference. **(b)** Measured feature dimensions for ASD's fabricated with three different concentrations (1.5%, 2%, and 3%). Each bar represents the mean measured dimension (*N* = 10) for each nominal feature width (0.5, 1, 1.5, and 2 mm), with individual data points overlaid. The numerical values on each bar indicate the mean feature dimension.

Feature fidelity was evaluated across devices fabricated with 1.5%, 2%, and 3% agarose (Figure 2). Concentrations below 1.5% failed to immobilize larvae, while >3% agarose yielded overly rigid devices. Across feature sizes from 0.5 to 2 mm, dimensional fidelity was maintained in all groups; however, 1.5% agarose demonstrated marginally superior accuracy for 0.5 and 1.0 mm features. Owing to this balance of flexibility, dimensional accuracy, and reduced material use, 1.5% agarose was selected as optimal for subsequent devices.

### 3.3 Optimization of slot geometry

**Figure 3:**
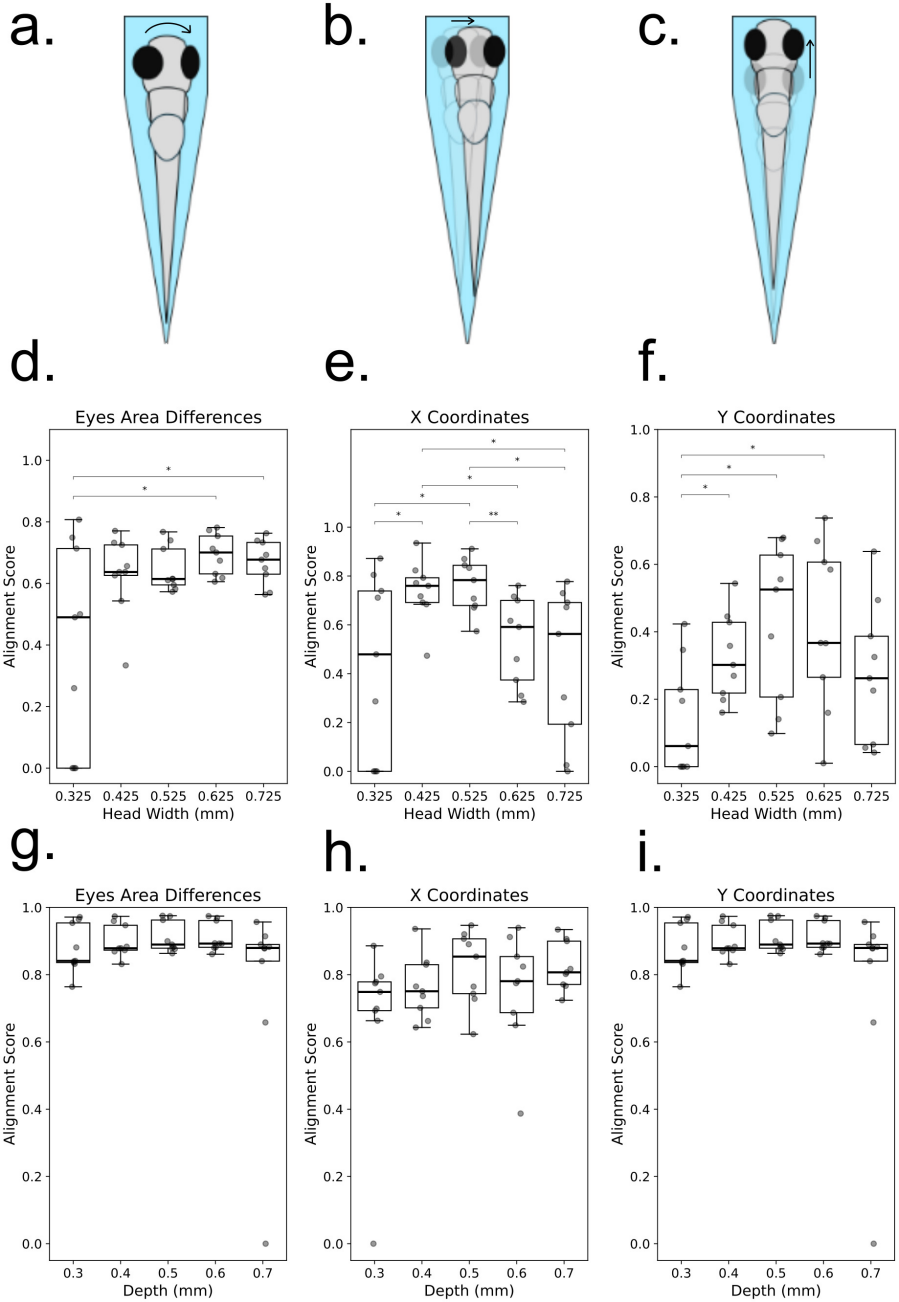
Influence of zebrafish larval positioning on alignment metrics in a microfluidic channel. **(a–c)** Schematic representations of common misalignments and instabilities observed when zebrafish larvae are positioned in a microfluidic channel: **(a)** Rotation along the anteroposterior (AP) axis, identifiable by asymmetrical eye positioning; **(b)** Mediolateral (ML) displacement, characterized by lateral shifts in eye positions along the x-axis; **(c)** Anteroposterior (AP) displacement, observed as shifts in eye positions along the *y*-axis. **(d–f)** Alignment scores for zebrafish larvae with different head widths at a fixed channel depth, quantified using three criteria: **(d)** Eye area asymmetry, where significant differences are observed between groups 1 and 4 (*p* = 0.02706) and groups 1 and 5 (*p* = 0.03910) (Welch's *t*-test); **(e)** *X*-axis displacement, showing significant differences for multiple comparisons: group 1 vs. 2 (*p* = 0.04048), group 1 vs. 3 (*p* = 0.02865), group 2 vs. 4 (*p* = 0.01505), group 2 vs. 5 (*p* = 0.02234), group 3 vs. 4 (*p* = 0.00664), and group 3 vs. 5 (*p* = 0.01471) (Welch's *t*-test); **(f)** *Y*-axis displacement, with statistically significant differences observed for groups 1 vs. 2 (*p* = 0.02648), groups 1 vs. 3 (*p* = 0.01294), and groups 1 vs. 4 (*p* = 0.01655) (Mann–Whitney *U*-test). **(g–i)** Alignment scores for zebrafish larvae with different depths at a fixed head width, assessed using the same three criteria: **(g)** Eye area asymmetry, **(h)** X-axis displacement, and **(i)** *Y*-axis displacement. Individual data points are displayed on each box plot, with statistical significance indicated.

Slot width and depth were systematically varied to assess larval alignment and stability (Figure 3). Alignment was quantified using eye area asymmetry, *x*- and *y*-coordinate variation, and stability metrics. Widths of 0.425–0.625 mm significantly outperformed narrower or wider slots (*p* < 0.05), with 0.525 mm providing the most consistent alignment. Depth variation was less pronounced, though 0.5 mm yielded optimal stability without excessive restriction. These values were adopted as default geometries for behavioral assays.

### 3.4 Free-tail and free-eye validation

**Figure 4:**
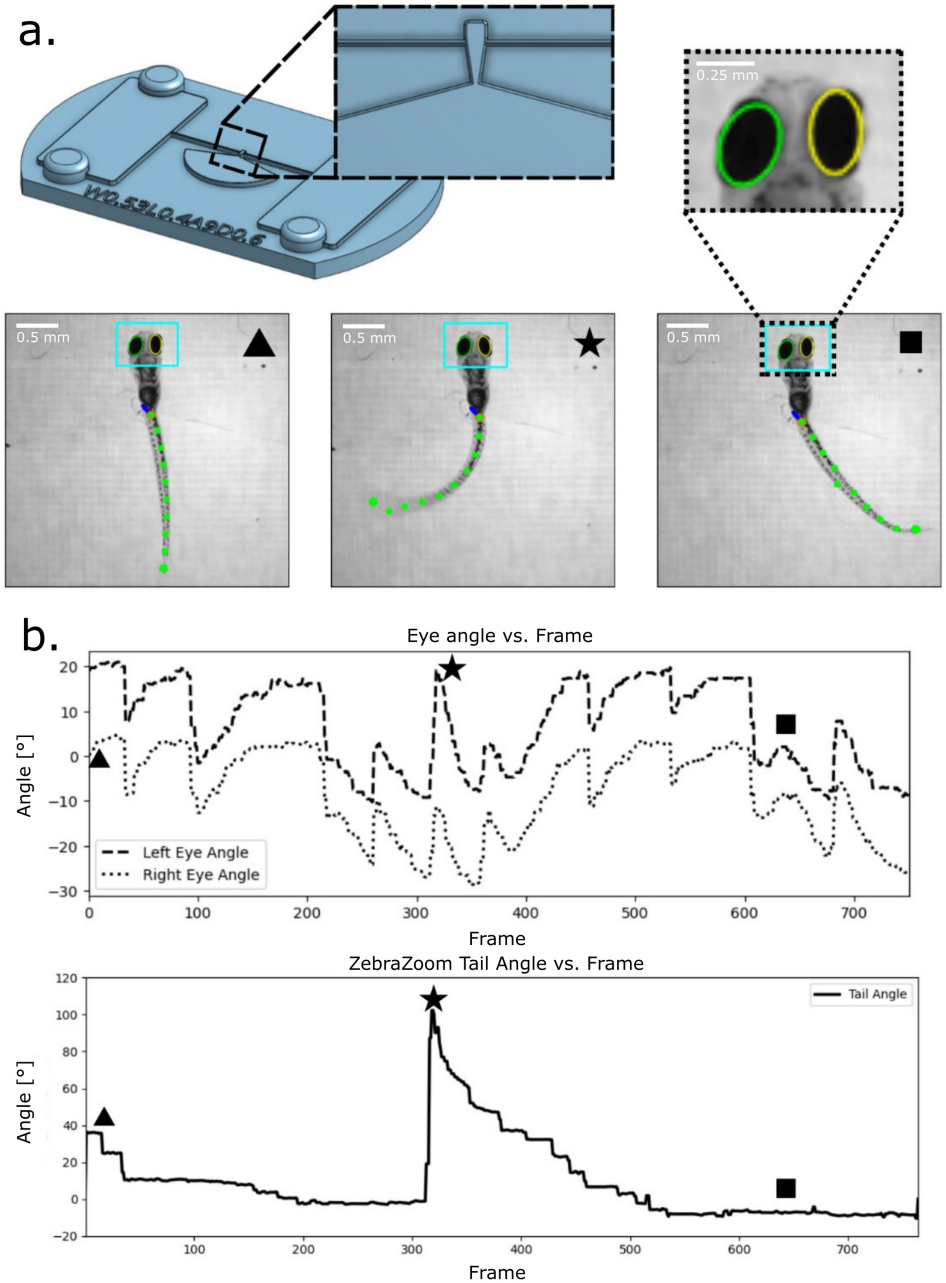
Larvae eye and tail tracking. A head fixed with free tail feature was designed to test eye and tail tracking with an optokinetic response protocol. **(a)** Device design and key frames from trial. A tapered section of the tail leads toward a wide cavity to fix the larva body while allowing for tail movement. A recorded video of this trial was passed through ZebraZoom for tail tracking (green dots on the tail) and that same video was ran through custom-made eye tracking software (teal bounding box). **(b)** The resulting eye tracking and tail tracking over time plots from our custom software and ZebraZoom respectively. Keyframes from **(a)** are displayed on the plots to display representative tail extrema and eye positions.

To evaluate the capacity for partial mobility, free-tail and free-eye configurations were designed (Figure 4). During optokinetic response (OKR) trials with rotating gratings, larvae immobilized in free-tail devices exhibited robust eye rotations aligned with stimulus direction and tail deflections corresponding to optomotor activity. Eye tracking was quantified using custom Python-based ellipse-fitting software, while tail movement was independently validated through ZebraZoom. Both pipelines detected stimulus-locked behavioral outputs, confirming that immobilization preserved natural motor responses while providing stability for imaging.

### 3.5 Feeding assays

**Figure 5:**
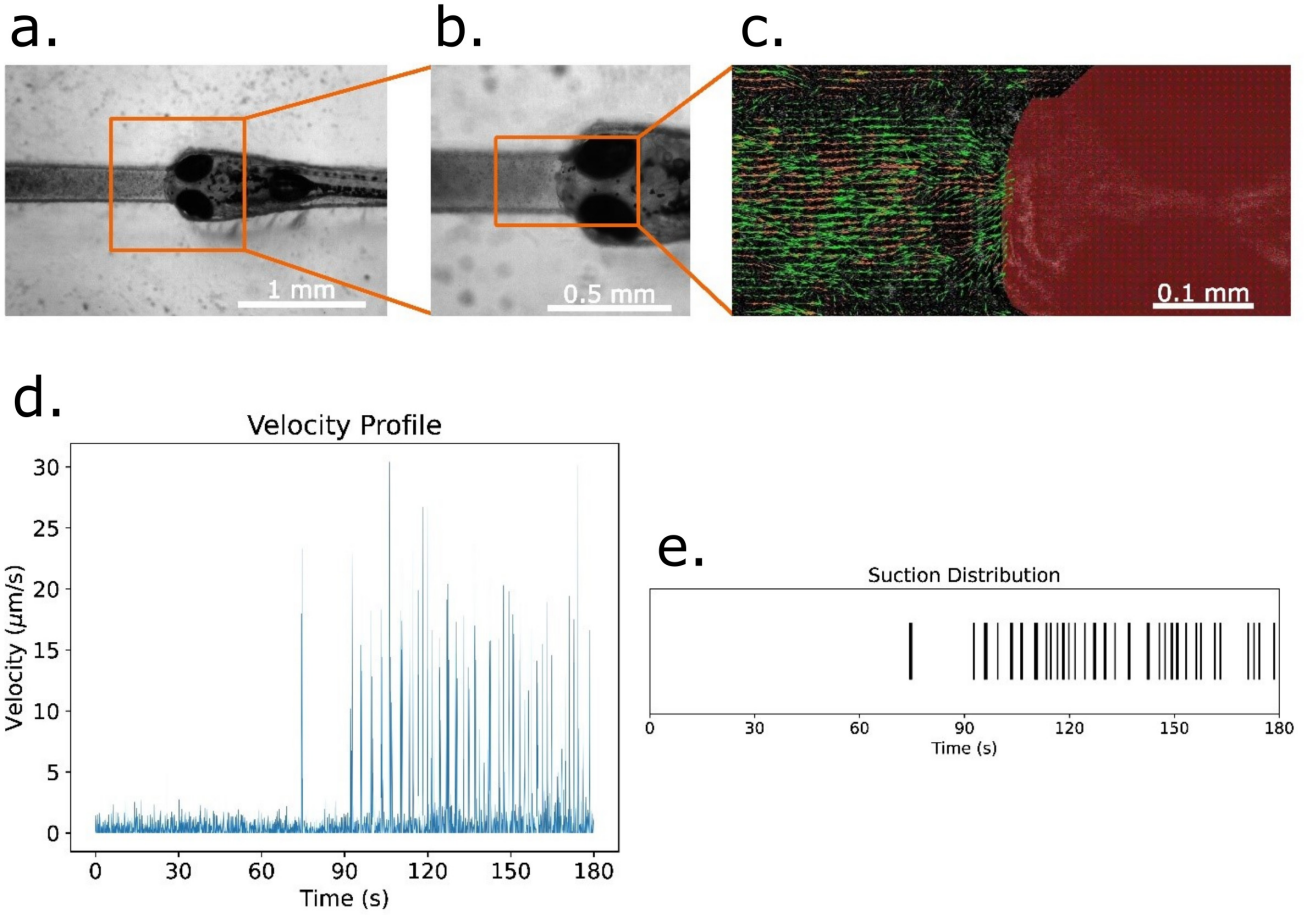
Analysis of food particle suction and velocity in zebrafish larvae. **(a)** Zebrafish larva immobilized within a microfluidic slot designed using an agarose stamping device for food-suction analysis. **(b)** Magnified view of the larva within the slot, highlighting its constrained position for controlled observations. **(c)** Particle Image Velocimetry (PIV) Analysis of food particle motion near the larva's mouth. Velocity vectors represent particle displacement within a single frame. **(d)** Temporal profile of the average velocity of food particles across all frames during a 3-min experiment. **(e)** Suction event distribution over time, extracted from velocity measurements by identifying peak velocity occurrences as suction events.

A free-mouth configuration was developed to study suction feeding. Food particles suspended in water were introduced into microchannels adjacent to immobilized larvae (Figure 5). Particle Image Velocimetry (PIV) revealed distinct suction events corresponding to larval ingestion attempts. Velocity profiles demonstrated temporally resolved peaks, with increased suction frequency in the later portion of recording sessions. These results establish the ASD as a platform for quantifying feeding kinematics under controlled conditions.

### 3.6 High-throughput screening

**Figure 6:**
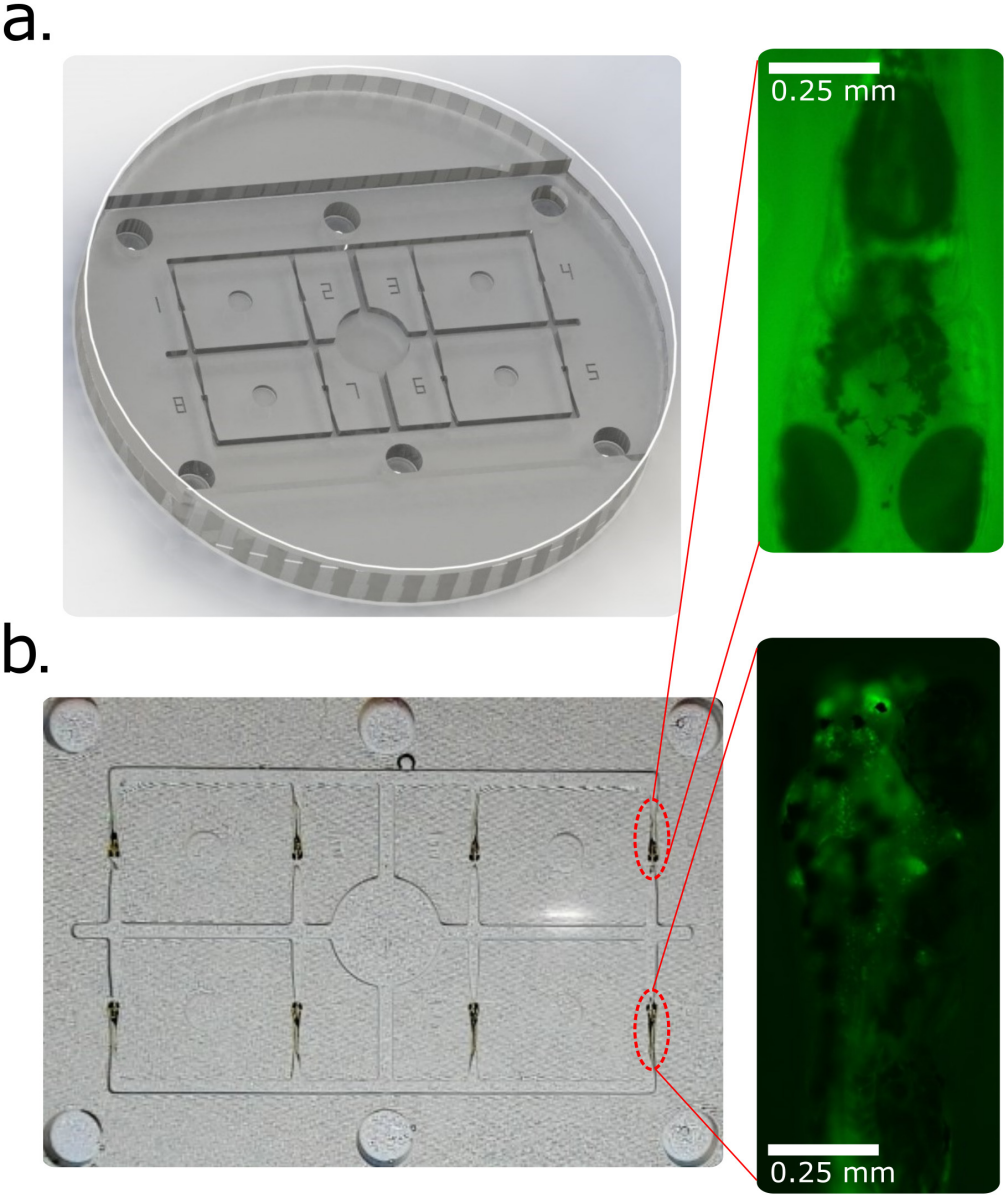
High-throughput screening of transgenic zebrafish larvae using the Agarose Stamped Device (ASD). **(a)** 3D-rendered CAD model of the zebrafish screening platform within a Petri dish, designed for parallel analysis of multiple larvae. The ASD facilitates rapid and reproducible positioning of larvae for fluorescence-based screening. **(b)** Screening of eight transgenic zebrafish larvae to distinguish fluorescently labeled individuals from non-expressing controls. The inset images highlight representative larvae: the upper inset shows a negative example (no detectable fluorescence), while the lower inset depicts a positive transgenic larva with fluorescent marker expression in motor neurons.

Transgenic larvae (4–6 dpf) were positioned in ASD cavities for fluorescence-based screening (Figure 6). Multiple larvae were immobilized simultaneously, enabling rapid identification of fluorescent-positive individuals. Because larvae were not fully embedded, retrieval was straightforward and survival rates were markedly higher than with conventional agarose embedding, which often damages specimens upon removal. This demonstrates the ASD's utility for efficient genotyping and transgenic line propagation.

### 3.7 Reusability and adaptability

Stamps and ASDs demonstrated reusability across multiple sessions. Agarose devices retained structural fidelity for weeks when kept hydrated. Stamp geometries could be adjusted to accommodate larval stages from 4–30 dpf, with modifications to cavity dimensions preserving immobilization across developmental growth. Collectively, these features promote reproducibility, scalability, and accessibility.

## 4 Discussion

### 4.1 Improving behavioral assays through standardized immobilization

The Agarose Stamped Device (ASD) eliminates many of the inconsistencies inherent in traditional agarose embedding by producing uniform larva-sized wells. This allows larvae to self-orient in consistent positions, reducing variability across experiments. Standardized immobilization is particularly valuable for assays such as the optokinetic response (OKR), where even small orientation differences can confound results. In this respect, the ASD provides a methodological foundation for behavioral neuroscience studies that require high reproducibility.

### 4.2 Balancing stability and natural behavior

A central finding of this work is that agarose concentration and cavity geometry strongly influence larval well-being and behavioral fidelity. Concentrations above 3% agarose produced rigidity that could restrict motor output and cavities that were too narrow caused excessive compression of the head. By contrast, the combination of 1.5% agarose, 0.525 mm slot width, and 0.5 mm depth struck an optimal balance between immobilization and freedom of movement for 6 days post fertilization (dpf) larvae. This is a critical point as immobilization methods must provide stability without silencing the very behaviors they are meant to help measure.

### 4.3 Expanding behavioral repertoires

The ASD permits behaviors that were previously inaccessible under immobilized conditions. Free-tail and free-eye configurations allow simultaneous tracking of visual and motor outputs, supporting integrated analyses of visuomotor coordination. Similarly, free-mouth configurations enable suction-feeding assays, providing a controlled way to examine ingestion dynamics that have ecological and metabolic significance. Importantly, the standardized slots and reservoir-supported design of the ASD enable elongated immobilization without inducing significant stress, thereby maintaining larval viability for extended experimental sessions. This stability allowed optokinetic response (OKR) activity to be tracked continuously for half an hour or longer using ASD in our Zebrafish Larva Interface platform (Jutoy et al., 2025). Together, these features broaden the behavioral repertoire available to researchers using the zebrafish larvae, extending beyond simple immobilization for imaging to include assays that capture naturalistic outputs over prolonged timescales.

### 4.4 High-throughput and screening applications

The potential of the ASD extends beyond individual assays. By arraying multiple larvae, researchers can conduct parallel experiments and efficiently screen for transgenic expression. This high-throughput capability addresses one of the major bottlenecks in zebrafish research: the time required to prepare and evaluate large numbers of larvae. Importantly, the open design of the ASD minimizes injury during retrieval, increasing survival rates compared to conventional embedding. These advantages make the ASD a practical solution for labs that require both scale and precision.

### 4.5 Cost efficiency and accessibility

The Agarose Stamped Device (ASD) provides a cost-effective alternative to both traditional and advanced immobilization strategies. Conventional agarose embedding requires little financial investment but is labor-intensive, low-throughput, and prone to variability in larval positioning. At the other extreme, microfluidic platforms offer precise spatial control and integrated stimulus delivery, yet they demand cleanroom fabrication, specialized expertise, and substantial expense, with device and infrastructure costs often reaching several hundred to thousands of U.S. dollars. Similarly, commercially manufactured molds for zebrafish larva immobilization typically cost several hundred dollars per unit and cannot be readily adapted once purchased.

In contrast, the ASD can be fabricated with a consumer-grade resin 3D printer (~$200–400) and low-cost resin consumables. The material cost for each stamp is negligible (on the order of cents), and once printed, stamps can be reused indefinitely to generate many agarose devices. Designs can be iterated or customized without additional investment, enabling laboratories to create a suite of stamps optimized for different larval stages or behavioral assays. Consequently, for the price of a single commercial mold, a laboratory can fabricate hundreds of ASD stamps, each tailored to evolving experimental needs. This combination of negligible per-stamp cost, scalability, and design flexibility substantially lowers the barrier for adoption in both resource-limited and well-equipped laboratories, while preserving the experimental advantages typically reserved for higher-cost platforms.

### 4.6 Current scope and future applications

While the present work demonstrated robust tracking of optokinetic responses and suction-feeding behavior, enabled by micrometer-scale resolution of eye, tail, and mouth movements, the methodology remains limited when extended to high-resolution neuroimaging. Specifically, although the ASD provides sufficient stability for prolonged behavioral assays, the current immobilization strategy does not yet achieve the submicron stability required for long-term optical recordings of larval brain activity. Addressing this limitation may require further optimization and exploration of stamp geometry features.

Despite these current constraints, the ASD highlights the value of methodological innovations that bridge simplicity and precision. By balancing stability with behavioral freedom, it enables experiments that were previously split between low-barrier agarose embedding and high-barrier microfluidic approaches. Future developments could expand the system to study collective or social behaviors by arraying larvae in interacting configurations, or to incorporate controlled microfluidic flow for assays such as rheotaxis. Thus, while the present device primarily advances individual-level behavioral tracking, it establishes a flexible foundation for both refined neurophysiological imaging and group-level behavioral assays. Notably, supplementary work confirms compatibility with water-immersion objectives typically employed for calcium imaging. By placing a hydrophobic coverslip above a larva in the cavity of the ASD and adding a water droplet above the sample, we demonstrated stable imaging with a 20 × /0.50w Olympus water-immersion objective, supporting the adaptability of the ASD for future neuroimaging applications (see Supplementary Figure 1).

## 5 Conclusion

The Agarose Stamped Device (ASD) offers a versatile, low-cost, and scalable platform that advances behavioral experimentation with zebrafish larvae. By enabling reproducible and gentle immobilization in standardized orientations, the ASD overcomes major drawbacks of conventional agarose embedding, particularly the inconsistent access to sensory and motor regions required for behavior assays.

Our findings show that customizable geometries permit fixation modes such as free-tail and free-eye configurations, which preserve natural eye rotations and tail movements essential for optokinetic and optomotor response assays. Similarly, free-mouth designs allow quantitative measurements of suction and feeding behaviors, while array formats streamline high-throughput screening of transgenic lines. These applications demonstrate that the ASD not only stabilizes larvae for imaging but also maintains the freedom of key motor outputs necessary to capture ecologically and experimentally relevant behaviors.

Beyond individual assays, the ASD supports simultaneous multi-larva alignment, enabling comparative studies across cohorts and integration with both custom and widely adopted analysis pipelines. Its modular design allows adaptation across developmental stages and experimental paradigms, lowering the technical threshold for laboratories with diverse research focuses.

In sum, the Agarose Stamped Device provides an accessible and refined solution for standardized larval immobilization that enhances the precision, throughput, and behavioral scope of zebrafish research. By balancing stability with behavioral freedom, the ASD expands the experimental repertoire available for investigating sensorimotor function, feeding dynamics, and developmental phenotypes *in vivo*.

## Data Availability

The datasets presented in this study can be found in online repositories. The names of the repository/repositories and accession number(s) can be found in the article/Supplementary material.
