# Supplementary material for: Agarose stamped method: a simple and customizable immobilization technique for zebrafish larvae
Source: Front Behav Neurosci. 2025 Oct 7;19:1692708. doi: 10.3389/fnbeh.2025.1692708 (PMC12537788; doi:10.3389/fnbeh.2025.1692708)
Supplement: Supplementary file 1 [file Supplementary_file_1.pdf]

Supplementary Figure 1: Compatibility of the agarose-stamped device (ASD) with water-immersion objectives.

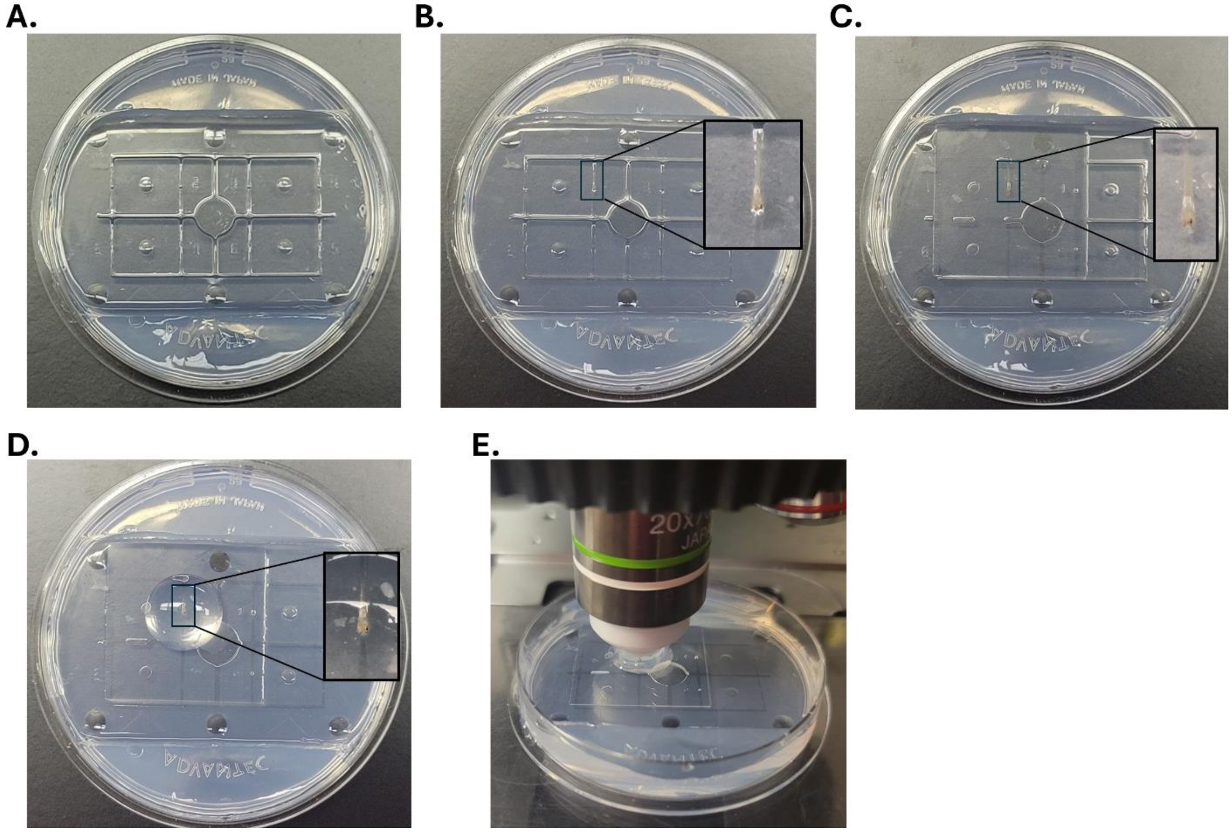

(A) An agarose stamped device used for screening applications. (B) Zebrafish larvae are transferred into the designated slot(s). (C) A 22 × 22 mm hydrophobic coverslip (Avantor, Cat. No. 48366-067) is placed over the sample. (D) A small drop of water is added on top of the coverslip directly above the specimen. (E) Imaging is performed using a water-immersion objective; in our experiments, a 20×/0.50w Olympus objective was employed.
