# Supplementary material for: Agarose stamped method: a simple and customizable immobilization technique for zebrafish larvae
Source: Front Behav Neurosci. 2025 Oct 7;19:1692708. doi: 10.3389/fnbeh.2025.1692708 (PMC12537788; doi:10.3389/fnbeh.2025.1692708)
Supplement: Supplementary file 2 [file Data_Sheet_1.pdf]

Supplementary Data and Repositories

Detailed protocols for the Agarose Stamping Method, including editable CAD files and video instructions, are available at: https://jjutoy2.github.io/Agarose-Stamping-Device/

All data and analysis scripts supporting this study are openly available at the following repository: https://github.com/JJutoy2/Agarose-Stamping-Device
